# Fluorescence Intrinsic Characterization of Excitation-Emission Matrix Using Multi-Dimensional Ensemble Empirical Mode Decomposition

**DOI:** 10.3390/ijms141122436

**Published:** 2013-11-14

**Authors:** Chi-Ying Chang, Chia-Chi Chang, Tzu-Chien Hsiao

**Affiliations:** 1 Institute of Biomedical Engineering, National Chiao Tung University, 1001, University Road, Hsinchu 30010, Taiwan; E-Mail: Deliachang.iie00g@nctu.edu.tw; 2 Institute of Computer Science and Engineering, National Chiao Tung University, 1001, University Road, Hsinchu 30010, Taiwan; E-Mail: ccchang.cs97g@g2.nctu.edu.tw; 3 Department of Computer Science, National Chiao Tung University, 1001, University Road, Hsinchu 30010, Taiwan

**Keywords:** excitation-emission matrix (EEM), multi-dimensional ensemble empirical mode decomposition (MEEMD)

## Abstract

Excitation-emission matrix (EEM) fluorescence spectroscopy is a noninvasive method for tissue diagnosis and has become important in clinical use. However, the intrinsic characterization of EEM fluorescence remains unclear. Photobleaching and the complexity of the chemical compounds make it difficult to distinguish individual compounds due to overlapping features. Conventional studies use principal component analysis (PCA) for EEM fluorescence analysis, and the relationship between the EEM features extracted by PCA and diseases has been examined. The spectral features of different tissue constituents are not fully separable or clearly defined. Recently, a non-stationary method called multi-dimensional ensemble empirical mode decomposition (MEEMD) was introduced; this method can extract the intrinsic oscillations on multiple spatial scales without loss of information. The aim of this study was to propose a fluorescence spectroscopy system for EEM measurements and to describe a method for extracting the intrinsic characteristics of EEM by MEEMD. The results indicate that, although PCA provides the principal factor for the spectral features associated with chemical compounds, MEEMD can provide additional intrinsic features with more reliable mapping of the chemical compounds. MEEMD has the potential to extract intrinsic fluorescence features and improve the detection of biochemical changes.

## 1. Introduction

Fluorescence spectroscopy plays an important role in the clinical detection of cancer tissue. The progression of cancer contains a series of complex changes, such as metabolic activity and protein expression of cancerous tissue that differs from normal tissue. Some of the chemical compounds, such as nicotinamide adenine dinucleotide (NADH), tryptophan, and collagen, are related to the changes in its progression. These compounds can be detected by their fluorescence properties [1]. Recently, fluorescence spectroscopy has been used as a non-invasive method for the detection of lesion tissue.

Traditional studies have used the difference in the fluorescence spectra between normal tissue and lesion tissue for disease discrimination, such as oral cancer [2] and cervical cancer [3]. Some studies determined specific spectral features that are associated with certain compounds in normal and lesion tissue, such as NADH and collagen [2,4,5]. To improve the feasibility and accuracy of fluorescence spectroscopy analysis, advanced feature-extraction methods, such as partial least-squares (PLS) analysis [5,6] and principal component analysis (PCA) [6–8], have been carried out at several excitation wavelengths to determine the principal components relevant to the disease. The complicated changes of the chemical compounds in tissue and photobleaching have hindered the determination of the wide mapping of chemical compounds in specific emission spectra. To obtain intrinsic information on the tissue constituents, excitation-emission matrix (EEM) was proposed as a characteristic mapping using a series of excitation wavelengths for specific emission spectral bands. To extract the features from EEM, multi-dimensional analysis methods have been applied, such as unfold partial least-squares (unfold-PLS) analysis [9], tri-linear partial least-squares (tri-PLS) analysis [9], and unfold principal component analysis (unfold PCA) [10]. Previous studies indicated that EEM features could be used as a model for tissue-type recognition. However, the spectral features of different tissue constituents were not fully separated by these linear multi-dimensional methods, and thus, the features of these constituents were not clearly defined.

PCA is widely used in fluorescence spectroscopy and EEM analysis. In this method, the correlations among principal components (PCs) are calculated and arranged in order of descending weights [11]. These PCs are uncorrelated to each other and contain most of the variability in the dataset. Generally, only the first few PCs are required to determine most of the variance in the original dataset, but the features related to tissue constituents may not be recognized in these PCs because the variance of several features may be similar and thus be presented in the same PC. In this case, features of different chemicals would not be separated. Moreover, conventional PCA extracts only those features along the direction of the emission wavelength (λ_emi_). For a more precise analysis of EEM, both directions of the excitation wavelength (λ_exci_) and λ_emi_ must be considered.

A novel adaptive decomposition method called ensemble empirical mode decomposition (EEMD) was recently proposed [12]. EEMD extracts the intrinsic component adaptively on multiple scales based on empirical mode decomposition (EMD), which was proposed by N.E. Huang. This approach is designed to seek the intrinsic characteristics of oscillations, termed the intrinsic mode function (IMF). EEMD is a revised version of EMD; it functions as a noise-assisted data analysis method and is a more consistent process than EMD [12]. EEMD overcomes some of the drawbacks of EMD, such as the mode-mixing problem. EEMD has been used for non-stationary analysis in many fields because it decomposes data efficiently. By decomposing data in each dimension, EEMD could be further extended to multi-dimensional ensemble empirical mode decomposition (MEEMD) [12], in which the intrinsic characteristics would be contained in the bi-dimensional intrinsic mode function (BIMF).

Several chemical compounds change in lesion tissues; however, fluorescence features that are related to these changes cannot be identified easily and with certainty. In this study, MEEMD was applied to biological EEM analysis. To control biological variation, fresh fish fillets were used as testing samples. Many important fluorophores, such as collagen type I, type V, and NADH, are related to the changes in fish during different stages [13]. The aim of this paper was to extract the intrinsic fluorescence characteristics that are related to the chemical constituents of tissue from EEM by using MEEMD and to compare its feasibility with the results from PCA.

## 2. Results and Discussion

The EEM of each fish sample was acquired at different storage times. Most EEM exhibited a major peak at excitation and emission wavelengths of 360 and 475 nm, respectively. Normalization was used to eliminate any differences between each measurement that may have been caused by unstable power output from the xenon lamp and the line scan property of the measurement system [14]. The intensity of each EEM was normalized before decomposition by the following equation:

(1) $$G(\lambda_{\text{exci}},\lambda_{\text{emi}})=\frac{F(\lambda_{\text{exci}},\lambda_{\text{emi}})}{F(\lambda_{\text{exci}},\lambda_{\text{emi}}+30)}$$

where λ_emi_ and λ_exci_ represent the emission and excitation wavelengths, respectively; *F* represents the original intensity of the EEM; and *G* represents the normalized intensity of the EEM [14]. Equation (1) was used to normalize the fluorescence intensity of each excitation-emission wavelength pair, *i.e*., *F*(λ_exci_, λ_emi_), by the fluorescence emission intensity at λ_exci_ +30 nm.

**Figure 1:**
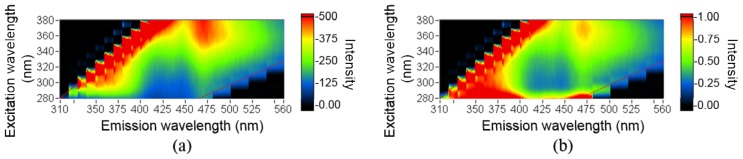
(**a**) Original excitation-emission matrix (EEM); (**b**) Normalized EEM. The collected data are between two oblique lines.

After normalization, a broad peak was observed at excitation and emission wavelengths of 340 and 475 nm, respectively (Figure 1). Additional small (λ_exci_, λ_emi_) peaks were observed at approximately (340, 400 nm), (280, 375 nm), and (280, 460 nm). Due to the complexity of tissue, its EEM presents several fluorescence features that are related to different chemical compounds. However, it is difficult to define the features of chemicals through the original and normalized EEMs.

### 2.1. PCA

#### 2.2.1. Main PCs

**Figure 2:**
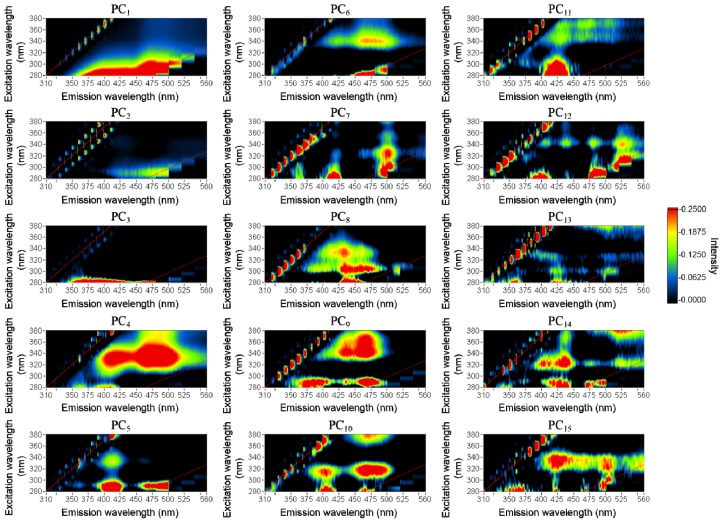
The first 15 principal components were considered the main components after modeling.

PCA was applied to fish EEM analysis at different storage times, and the main PCs were selected after modeling. Through leave-one-out cross-validation, the root mean square error (RMSE) of the prediction results for different modeling components were obtained (see details in Section 3.4.1). The model that contained the first 15 components had the smallest RMSE. Thus, the first 15 PCs were considered the main PCs (Figure 2). Each PC provides information along the direction of λ_emi_, which is related to changes in the chemical compounds within the tissue.

#### 2.2.2. Relationship between PCs and the Chemical Compounds

Fish tissue contains many types of chemicals, e.g., collagen, tryptophan, and lipids. The amount of each chemical and its changes during storage differ. Although the first few PCs describe the largest variance of the EEM, these PCs exhibit a combination of features that have the largest variance. Some of the chemical-related features would not be responsible for the largest variance in the dataset and would thus not be represented in these initial PCs. The other PCs are likely more related to chemical features. Because most of the chemicals in fish tissue change during storage, we calculated the mapping coefficient, b, to examine the relationship between PCs and storage time. After singular value decomposition, dataset (**X**) was converted into three matrices, **U**, **S**, and **V. U** contains the eigenvectors of **XX**^T^, **S** contains the squared roots of the eigenvalues of both **XX**^T^ and **X**^T^**X**, and **V** has the eigenvectors of **X**^T^**X**. The mapping coefficient, **b** = (**P**^T^**P**)^−1^**P**^T^**y**, in which **P** = **US** and **y** denotes the storage time of the amberjack, was calculated by using least squares (see Section 3.4.1 for details). The PCs that have larger b values would be related to chemical changes during storage. The b value of each PC was calculated, and all of the PCs were rearranged by descending b values. The first five PCs were selected as the main components. We also compared the feature locations with the references listed in Table 1[1,4,5,13,15,16] and found that PCs with larger b values present features related to chemicals. The main components are PC_14_, PC_15_, PC_12_, PC_11_, and PC_8_ in order of descending *b* values. PC_14_ displayed a feature at approximately (290, 440 nm), which was related to NADH. There were also two peaks related to collagen, which were located at (325, 400 nm) and (330, 430 nm) [13]. PC_15_ displayed features at (280, 350 nm), which was related to tryptophan [15], and at (330, 430 nm), which was related to collagen [13,16]. PC_12_ displayed a feature that was related to NADH at (350, 450 nm) [1,4,5]. PC_8_ had features of NADH and collagen that were located at (290, 440 nm) and (330, 430 nm), respectively [1,13]. There were several features related to other chemical compounds in the tissue, such as adenosine triphosphate (ATP), lipids, and vitam**i**ns. The fluorescence features of the tissue constituent are listed in Table 1[1,4,5,13,15,16].

### 2.2. MEEMD

**Figure 3:**
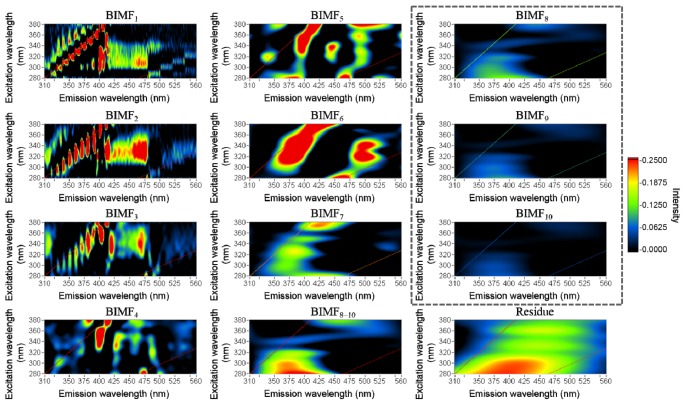
The first 10 bi-dimensional intrinsic mode function (BIMF) were considered important after modeling. BIMF_8_–BIMF_10_, which are in the dashed box, were combined as BIMF_8–10_.

MEEMD was applied to the fish EEM individually (see Section 3.4.2). Ten BIMFs and one residue were obtained. Each BIMF contains the features of each spatial variable scale in the EEM. BIMF_8_–BIMF_10_ were combined based on their intrinsic feature similarities. The results of the BIMFs were consistent in each EEM at each time. Figure 3 presents 10 BIMFs and the combination of BIMF_8–10_ at one sample as an example. BIMF_3_–BIMF_6_ provided the intrinsic information related to the tissue constituents. A previous study demonstrated that the changes of the EEMs at (330, 430 nm) and (330, 470 nm) were correlated to type I and type V collagen, respectively [13]. In BIMF_3_, the changes of the EEM located at (330, 430 nm) and (330, 470 nm) may be related to collagen, which is an important compound in fish tissue. Conventional studies investigating the fluorescence peaks at (340, 455 nm) and (290, 460 nm) indicated that the peaks were due to NADH and could be considered important features in the determination of different storage times of fish [13,16] and for distinguishing between lesion and normal tissue [1]. The peaks at (340, 460 nm) in BIMF_4_ and at (290, 460 nm) in BIMF_6_ may be related to NADH [1,4,5]. BIMF_5_ displayed a peak located at (275, 358 nm), which is related to tryptophan [15]. The fluorescence features contributed by the chemical compounds are listed in Table 1. In addition, the changes in features during storage were examined. The spectral distribution of each fluorophore would differ due to changes in the peak intensity and in the intensity of the main emission region. To examine the changes in the spectrum, the area under the spectrum was integrated and used instead of the peak intensity. The feature of the BIMFs would more likely be related to the chemicals if they changed during storage and the change exhibited a similar pattern in terms of chemical changes. The main emission region of collagen is at (310–350, 410–440 nm) in BIMF_4_, and the significant spectral peak of this intrinsic feature was located at (335, 429 nm) after averaging for different storage times. The integration values decreased during storage, and the correlation coefficient of the integrated value and storage time was −0.81, which indicated that the extracted intrinsic feature was likely related to collagen.

### 2.3. Comparison between MEEMD and PCA

To understand tissue composition, chemical features must be extracted from the spectra. PCA decomposes EEMs and reveals their features; however, these features are presented by their variance and may not be related to the chemicals. In contrast, MEEMD could be viewed as a filter that can separate intrinsic oscillations having different frequencies [12]. BIMFs report fluorescence features and are arranged by their spatial frequency. In this study, BIMF_1_–BIMF_3_ were similar, but these features were considered to be contributed by different fluorophores because the spatial frequency of each BIMF was different. In fact, the features that appeared at a similar location for different BIMFs were more likely the separation of overlapping features. MEEMD was applied to every EEM, and the corresponding BIMFs were similar. The fact that all of the EEMs yielded similar BIMFs indicates that each feature in the corresponding BIMFs was contributed by the same factor. The integration values of several features decreased during storage, which coincided with the chemical changes. It was unlikely that noise effects would appear at the same location for every EEM and decrease during storage. In this case, the features in the BIMFs were considered to be related to the chemicals. Thus, MEEMD can extract and separate chemical features from EEMs.

PCs and BIMFs were extracted from normalized EEM as the intrinsic features related to the tissue constituents, such as NADH, collagen, and tryptophan. Some of the intrinsic features were obtained in the PCs and BIMFs. Although these intrinsic features may present changes in the tissue constituents during cancerous processes, several features have not been clearly identified. These features might be related to chemical compounds that are unknown and must be addressed in the near future. There are also several unexpected peaks in the upper-left portion of the BIMFs, which is outside the range of the measurements. This unexpected result may be due to the process of EEMD and the high-intensity fluorescence signal acquired at the beginning of the measurements. These unusual peaks would have to be eliminated for a complete assessment of EEM.

The features of different chemical compounds were not entirely separated in the PCA. One main component might contain multiple features and be related to several chemical compounds. Furthermore, the PCA results were different when the number of samples or variables was varied, which is a limitation of this method. This lack of consistency makes PCA unreliable for clinical applications. In contrast, BIMFs were extracted without modeling by MEEMD and yielded consistent fluorescence features of important chemical compounds. Moreover, a single BIMF presented individual intrinsic features in EEM, and the features presented by BIMFs could be linked to specific chemical compounds. Overall, MEEMD demonstrated its utility in the detection of chemical compounds by fluorescence feature extraction from EEM. MEEMD provides an effective approach for EEM analysis and for non-invasive tissue diagnosis.

## 3. Experimental Section

### 3.1. Sample

Fresh fish fillets were used as the experimental sample as a control for biological variations. Eighteen amberjack (*Seriola Dumerilli*) were used in this study [17]. Each amberjack weighted 1 ± 0.2 kg. After slaughter, the fish were washed with distilled water and sliced into specimens (4 × 3 × 1 cm) from the abdomen and dorsum. The samples were stored at 4 °C in a refrigerator.

### 3.2. Instruments

**Figure 4:**
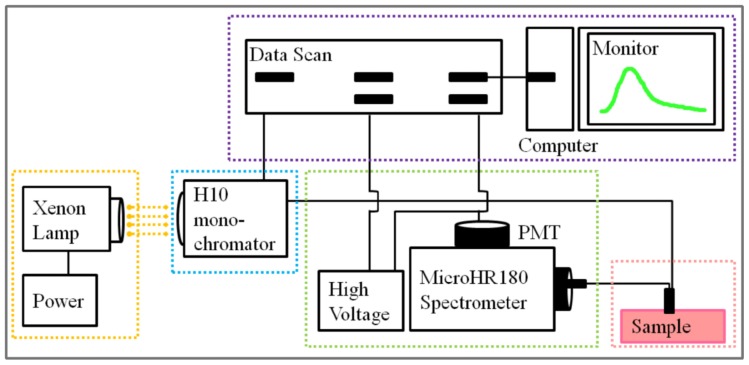
Measurement system.

A Y-type fiber optic measurement system was used for the fluorescence spectroscopy measurements in this study (Figure 4) [17]. This system contained a CERMAX^®^ xenon lamp (Perkin Elmer, Waltham, MA, USA), H10 monochromator (HORIBA Jobin Yvon, France), MicrHR180 spectrometer (HORIBA Jobin Yvon, France), R928 photomultiplier tube (PMT) (Hamamatsu, Japan), Y-type optical fiber (Oriel, Stratford, CT, USA), and general commercial desktop (ASUS, Taipei, Taiwan).

Broadband light was produced by a xenon lamp and was passed through an H10 monochromator to generate specific wavelengths within a narrow band. The samples were excited by the light at specific wavelengths, and the emitted fluorescence signals were collected. Because the surface of the fiber bundle was vertically oriented with respect to the samples, the emission signals were collected through the fiber bundle and split by the MicrHR180 spectrometer. The intensities of specific emission signals were acquired by the PMT. The controlling and acquiring processes were designed and implemented by commercial development software (LabVIEW 7.1, National Instruments Corp., Austin, TX, USA).

### 3.3. Procedure

The fish fillets were removed from the refrigerator for fluorescence measurement every 2 h for 24 h. The excitation wavelength (λ_exci_) was set from 280 to 380 nm with 10 nm increments, and the emission fluorescence intensity was measured from λ_exci_ + 30 nm to 2λ_exci_ − 80 nm. The intensities of the emission spectra acquired for each specific λ_exci_ were recorded and combined into the EEM. The total number of EEMs was 234 (18 amberjacks × 13 measurements of each amberjack during storage). The intrinsic features of the EEMs were then extracted by PCA and MEEMD and were reported as PCs and BIMFs, respectively. All of the analysis programs in this study were developed through commercial development software (LabVIEW v.2011, National Instruments Corp., Austin, TX, USA).

### 3.4 Analysis

#### 3.4.1. Main PCs by Unfold-PCA

**Figure 5:**
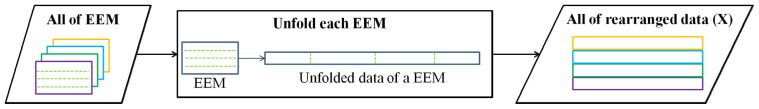
Rearranging procedure of the dataset.

Unfold-PCA is an extended method of PCA in which three-dimensional data are arranged into two-dimensional data prior to decomposition. In this study, the three-dimensional data for all of the samples with the dimensions (*n × m × l*) (where *n* denotes the number of EEM, *m* denotes the number of used λ_exci_, and *l* denotes the number of collected λ_emi_) were rearranged into a two-dimensional matrix, **X**, with dimensions of (*n × ml*) (Figure 5). Each row of **X** corresponds to an EEM. PCA could be achieved through SVD [11]. After data rearrangement, the PCs of **X** were obtained through SVD [11] using Equation (2).

(2) $$X={\mathrm{USV}}^{T}$$

**U** contains the eigenvectors of **XX**^T^, **S** contains the singular values, or squared roots of eigenvalues, of **XX**^T^ (or **X**^T^**X**), and **V** contains the eigenvectors of **X**^T^**X**. The eigenvectors and singular values are arranged in descending order of the singular value. In this study, each column vector of **V** was also considered a PC. The first few PCs generally represented the variation within the dataset. For EEM data, the initial PCs contain features related to the combination of the chemical compounds that have large variances. However, some compounds that have small variances would not be shown in these PCs. More components should be considered to obtain PCs related to chemicals. Principal component regression (PCR) with a leave-one-out cross-validation (LOOCV) procedure was adopted for the main PC selection. In the LOOCV procedure, the entire dataset **X**, which contains 234 EEMs obtained from 18 amberjacks with 13 storage times, were divided into two groups: training data (**X***_i_*) and testing data (**x***_i_*). **x***_i_* denotes the *i*^th^ EEM data, and **X***_i_* denotes the other 233 EEMs. Each **X***_i_* was decomposed by PCA to obtain **U**, **S**, and **V** to calculate the predicted y of **x***_i_*. The value of *i* ranged from 1 to 234. A time label (y*_i_*) was assigned to each **x***_i_*. This label was defined according to the chemical compounds in fish tissue that would change during storage. The amount of these chemical compounds in fish would decrease during its storage. y was defined as 1 for the data obtained at storage times of 0–12 h and 0 for the data obtained at storage times of 14–24 h. Because **XV = US**, the following regression formula was used:

(3) $$Y_{(n-1)x1}=P_{(n-1)\mathrm{xr}}b_{\mathrm{rx}1}+e_{(n-1)x1}$$

where **P**_233xr_ = **U**_233xr_**S**_rxr_, the number of training EEMs was 233, and r is the number of selected eigenvectors. The dimensions of **P** and **U** are 233 × *r*, and the dimensions of **S** are *r* × *r*. **b_rx1_*=*(P^T^P)**^−^**^1^P^T^y**, where **y** denotes the time labels of each EEM, **b**_rx1_ denotes the mapping coefficient calculated by using r eigenvectors, and the dimensions of **b** are *r* × 1. r contains 233 elements. **e** represents the error, and the dimensions of **e** are 233 × 1. Each **X***_i_* was converted into a set of **U**, **S**, and **V** matrices. Each set of **U**, **S,** and **V** was selected from *r* = 1–233. The corresponding **x**_i_ was used to obtain the predicted **y** (**ŷ**) according to **ŷ** = **Pb**, where **P** was obtained by **P** = **x***_i_***V**. The RMSE was calculated after all of the predicted y values were calculated by using different **x***_i_* values and a different number of eigenvectors. The optimal value function for the main PC selection was set as the minimal RMSE. Furthermore, the main components were arranged in order of descending b value, and the first five main components were considered the most important intrinsic components.

#### 3.4.2. MEEMD

**Figure 6:**
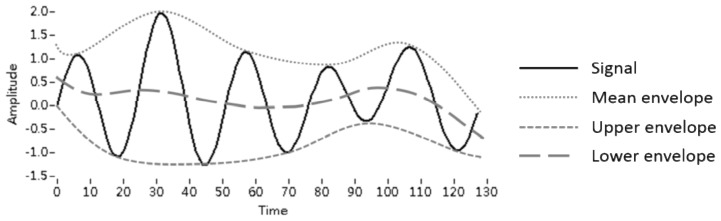
An example of envelopes.

MEEMD [12] was adopted to extract the intrinsic fluorescence features from the EEMs. The procedure of EMD is as follows: (i) find all of the local extrema for the source signal *x(t)*, which denotes the fluorescence spectrum in this study, and calculate the upper and lower envelopes with the maxima and minima, respectively, by cubic spline interpolation (Figure 6 provides an example of the envelopes); (ii) obtain the mean envelope from the average of the upper and lower envelopes; (iii) determine the intermediate component *h* by subtracting the source signal and mean envelope; (iv) treat the intermediate component *h* as the source signal and repeat steps (i)–(iii) until the mean envelope reaches 0 and the final component *h* is treated as an intrinsic component *c_j_*, also called the *j*^th^ IMF; and (v) take the difference between the original data and component *c_j_* as the source signal and repeat steps (i)–(iv) until there is no IMF to be extracted, treating the remainder as the residue *r*. The results are expressed by Equation (4).

(4) $$x(t)=\sum_{j=1}^{n}c_{j}+r$$

The EEMD procedure is as follows: (i) generate a series of white noise that has the same size as the data with a standard deviation of 0.2; (ii) add the white noise to the source signal before EMD; (iii) decompose the source signal with white noise into IMFs using EMD; (iv) repeat steps (i)–(iii) with different white noise levels; and (v) calculate the average of all of the corresponding IMFs and residues as the final IMFs in the EEMD process.

**Figure 7:**
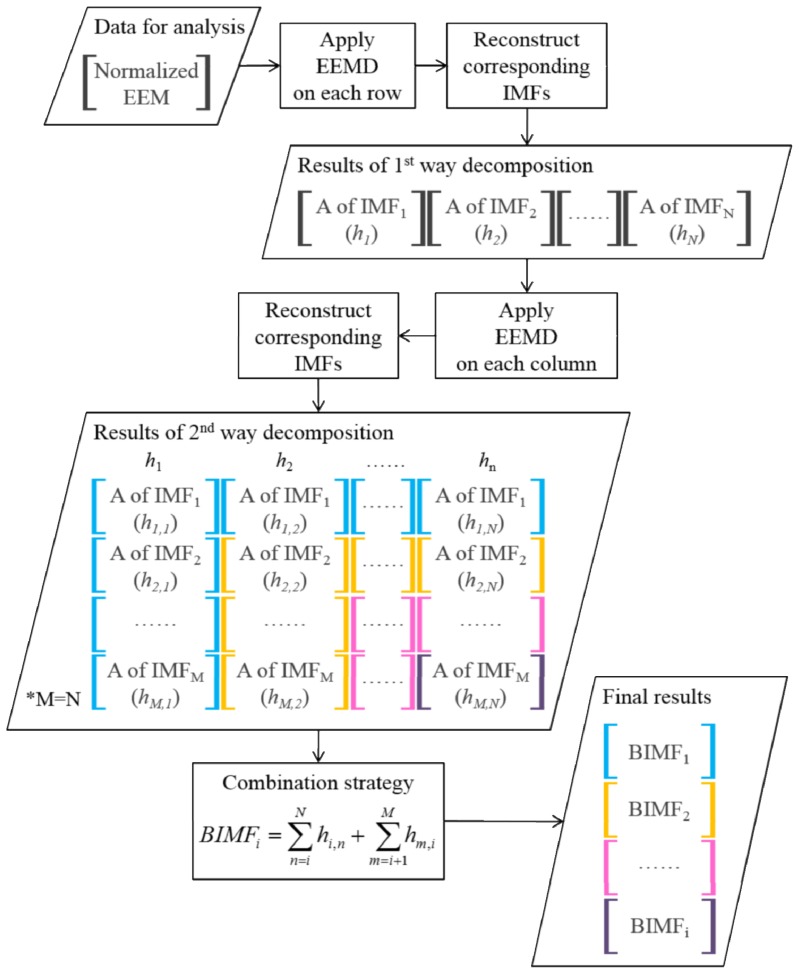
Multi-dimensional ensemble empirical mode decomposition (MEEMD) procedure.

EEMD was also applied to the multi-dimensional data by decomposing the source signal along each direction. The MEEMD procedure for EEM as two-dimensional data was as follows: (i) use EEMD to decompose the EEM along each excitation wavelength direction (row) in ascending order and obtain the first-stage IMFs; (ii) reconstruct the corresponding IMFs into two-dimensional data as the decomposition results in the first stage; (iii) use EEMD to decompose the results of step (ii) iteratively along the emission wavelength’s direction (column) in ascending order and obtain the second-stage IMFs; (iv) reconstruct the corresponding IMFs of the second stage into two-dimensional data as the decomposition results in the second stage; and (v) obtain the final results, *i.e*., BIMFs, by combining the IMFs that have comparable minimal scales. The MEEMD procedure is also shown in Figure 7.

## 4. Conclusions

The aim of this study was to examine the ability of MEEMD to extract intrinsic features from EEMs and to understand how these features are related to chemical compounds. PCA is a well-studied method and extracts the main features of the variance of source data. Because the PCs present the data variances, which are determined by their weighting, the features in the PCs are the combination of several data features. The features in the EEM, which are related to chemical compounds, were complicated, and some of the features had small variances. Thus, the main features related to chemical compounds were not entirely separated, and the PCs related to chemical changes were difficult to identify without modeling. In contrast, MEEMD can extract the intrinsic oscillations at the adaptive spatial frequencies and is a reliable method for EEM analysis on multiple spatial scales. The results demonstrated that MEEMD can extract fluorescence intrinsic features from an EEM without training. Moreover, the BIMFs extracted by MEEMD presented consistent features related to the chemical compounds, and the intrinsic features can fully describe the characteristics of the original EEM and normalized EEM. Future research will examine such topics as the identification of the relationship between the intrinsic features of the BIMF and the storage time of the fish as well as an investigation of the unknown features of the chemical compounds. Recent studies have demonstrated that other feature extraction methods, such as PLS and parallel factor analysis (PARAFAC), can extract the features from fish EEMs. A comparison of the performance and capability of these methods and MEEMD warrants further investigation. MEEMD allows us to obtain intrinsic spectral features of EEM along the directions of λ_exci_ and λ_emi_. This study suggests that MEEMD improves EEM analysis and provides a reliable adaptive view for EEM exploration of fluorescence spectrum diagnosis on multiple spatial scales.

## Figures and Tables

**Table 1:** Fluorescence features of the endogenous florophores.

Chemical compounds	Peak location (λ_exci_, λ_emi_) (nm)
Collagen	(325, 400), (330, 430), (330, 475)

Enzymes and coenzymes	
NADH	(290, 440), (350, 450)
FAD (Flavin adenine dinucleotide)	(368, 532)

Globin	(295, 340)

Amino acids	
Tryptophan	(280, 350)
Tyrosine	(275, 300)

ATP	(300, 400)

Lipids	(340–395, 430–460 and 540)

Vitamins	
Vitamin A	(327, 510)
Vitamin D	(390, 480)
Vitamin K	(335, 480)
Vitamin B_6_ compounds	(315–340, 385–425)
